# Climate change and mental health. Polish perspective

**DOI:** 10.1192/j.eurpsy.2024.204

**Published:** 2024-08-27

**Authors:** M. Gawrych

**Affiliations:** Psychology Institute, The Maria Grzegorzewska Univeristy, Warsaw, Poland

## Abstract

**Introduction:**

World Health Organization estimates that climate changes are expected to cause an additional 250 000 deaths worldwide per year between 2030 and 2050 (1). We do not know, in what extense, population mental health will deteriorate due to climate change. Unfortunately, not all European countries, including Poland collect the evidence-based information about current and possible future risks for mental health.

(1)World Health Organization. Climate change and health; 2018. Available from who.int/news-room/ fact-sheets/detail/climate-change-and-health [cited 20 October 2020]

**Objectives:**

The aims of present study are: (1) summarize the available literature through a current review and (2) make recommendations for future actions/ prevention strategy for Poland.

**Methods:**

Medline database (through PubMed) and Polish authorities documents was searched for records published in 2010–2024. Mental health-related descriptors (i.e. „mental health” OR “mental disorders”) and „climate change” and “Poland/ Polish” and “Europe” term were used in particular searches. The results of the screening were included in the final selection list. References of screened full-text articles and raports were manually searched for further literature. Additionally, European and worldwide publications and reports prepared by mental health and/ or climate change organizations were taken into account.

**Results:**

Mental health risks of climate change can stem from climate-related natural disasters (e.g., extreme weather events), slower moving events (e.g., drought), or concern about the phenomenon of climate change itself. Primary mental health impact is related mostly to disasters itself and its consequences: environment of disruption, trauma and grief. Direct consequences include increased rates of high-risk behaviours. Secondary effects of climate change are due to various processes of environmental changes and ecological disruptions. They consist of damages to physical and social infrastructure, physical health effects, food and water shortages, conflict, and displacement. Long-term droughts affect food and water supplies and can subsequently affect the economic and mental wellbeing not only the land-based workers.

**Conclusions:**

A focus on climate change impact on mental health can help enhance the understanding of factors that strengthening psychosocial resilience and adaptation. The future mental health challenges of climate change in Poland cover:

- developing scientific knowledge regarding adaptation process and resilience,
- focusing on high-risk groups (i.e. children, rural workers),
- strengthened community engagement,
- developing available locally strategies for mental health improving.

**Disclosure of Interest:**

None Declared

